# Resveratrol as a Potential Platelet Inhibitor in Aspirin-Resistant Diabetic Patients—A Novel Therapeutic Strategy Targeting F_0_F_1_-ATP Synthase Inhibition

**DOI:** 10.3390/life15111718

**Published:** 2025-11-06

**Authors:** Isabella Panfoli, Lavinia Carlini

**Affiliations:** 1 Department of Pharmacy (DIFAR), University of Genoa, 16132 Genova, Italy; 2 Faculty of Agricultural, Environmental and Food Sciences, Free University of Bozen-Bolzano, 39100 Bolzano, Italy; lavinia.carlini@unibz.it

**Keywords:** ATP synthase, aspirin resistance, oxidative stress, platelets, resveratrol

## Abstract

In Diabetes Mellitus (DM), a metabolic disorder characterized by elevated blood glucose due to impaired insulin action, platelet function is dysregulated and contributes to the pathological progression of the disease. In type 2 diabetes mellitus (T2DM), hyperglycemia, insulin resistance, oxidative stress, and inflammation impair endothelial function and platelet regulation, promoting a prothrombotic state. Platelet hyperreactivity is associated with T2DM cardiovascular complications, a leading cause of mortality in patients. Antiplatelet therapies often prove ineffective for a subset of T2DM patients due to aspirin resistance, necessitating alternative therapeutic strategies. Resveratrol, a natural polyphenol, is a potential therapeutic agent for T2DM, including inhibition of platelet aggregation. One of the pleiotropic actions of resveratrol is to modulate the F_o_F_1_-ATP synthase rotational catalysis. Platelet chemical energy demand during the activation phase is achieved through oxidative phosphorylation. Both mitochondrial and extra-mitochondrial oxidative phosphorylation drive aerobic energy production in activated platelets, utilizing fatty acids and glucose, respectively. Hyperglycemia can cause an overwork of the oxidative phosphorylation, producing oxidative stress. Targeting F_o_F_1_-ATP synthase with resveratrol may reduce platelet hyperreactivity in aspirin-resistant cases. This paper reviews the implications of resveratrol ability to inhibit platelet F_o_F_1_-ATP synthase on its potential as a novel alternative or synergistic antiplatelet strategy for aspirin-resistant T2DM patients.

## 1. Introduction

Diabetes mellitus (DM) is classified into type 1 and type 2. Type 1 DM (T1DM) is an autoimmune disorder defined by the destruction of pancreatic β-cells by autoreactive T lymphocytes, leading to absolute insulin deficiency [1]. Type 2 diabetes mellitus (T2DM), a metabolic disorder that presents with chronic hyperglycemia and an inadequate response to circulating insulin by peripheral tissues (insulin resistance), accounts for approximately 90% of global cases [2]. The growing prevalence of T2DM and its complications worldwide, both in high-income and low-income countries, is a significant public health challenge [2,3]. The global number of people with diabetes (primarily T2DM) is projected to exceed 1.3 billion by 2050 [3]. According to a system dynamics modelling study using national survey data, the population with diabetes in China, which has the highest number of patients with DM worldwide, is projected to face a dramatic growth in individuals with both DM (202.84 million by 2050) and cardiovascular disease (CVD) (122.88 million by 2050), representing a substantial future economic burden [4]. Unhealthy lifestyle choices and genetic predisposition are the primary causes of type 2 diabetes mellitus (T2DM), which is marked by the progressive loss of pancreatic β-cell function, leading to hyperglycemia [5]. Chronic oxidative stress and low-grade inflammation activate kinases, including NF-κB, thereby worsening insulin resistance and promoting β-cell apoptosis [6]. Impaired insulin signaling is driven by defects in the insulin receptor substrate (IRS)–phosphatidylinositol-3-kinase (PI3K)-Akt pathway in tissues (skeletal muscle, liver, and adipose tissue) and by mitochondrial dysfunction and endoplasmic-reticulum stress [7,8]. T2DM predisposes to both microvascular (diabetic retinopathy and nephropathy) and macrovascular (peripheral, coronary, and cerebral artery disease, atherosclerosis, and kidney disease) complications [9]. Cardiovascular complications are the leading cause of T2DM morbidity and mortality [5]. Increased cardiovascular risk in patients with type 2 diabetes mellitus (T2DM) is associated with structural platelet abnormalities and the presence of circulating immature platelets with mitochondrial dysfunction [10]. In T2DM, there is suppression of anticoagulant molecules, such as thrombomodulin, and impaired fibrinolysis, resulting in increased levels of circulating pro-inflammatory cytokines (TNF-α, IL-1, IL-6), pro-coagulant factors (von Willebrand factor, VWF), plasma fibrinogen, and thrombin [11]. Hyperglycemia, hyperlipidemia, low-grade inflammation, and oxidative stress contribute to platelet hyperreactivity, promoting a pro-thrombotic state in type 2 diabetes mellitus (T2DM). The inflammatory, pro-thrombotic environment heightens the cardiovascular risk observed in T2DM [12]. Therefore, in clinical practice, glucose-lowering, lipid-lowering drugs, and antiplatelet agents are employed [13,14]. By modulating platelet aggregation, it is possible to lower the risk of CVD [15].

Resveratrol (RSV) (3,4′,5-trihydroxy-*trans*-stilbene) is a polyphenolic phytoalexin structurally related to stilbenes consisting of two phenolic rings bonded by a double styrene bond [16] RSV is synthesized in considerable amounts in grapes, peanuts, berry fruits, and a variety of medicinal and edible plants in response to stress conditions [17]. The low RSV solubility affects absorption, which differs depending on the dietary source. After oral intake, RSV is rapidly absorbed in the small intestine through passive diffusion and binding to transporters. These include multidrug resistance–associated proteins MRP2 and MRP3, members of the ATP-binding cassette (ABC) transporter family, as well as integrins and others [18]. In humans, more than 70% of orally administered RSV is absorbed and rapidly metabolized (in less than 30 min). Its half-life is about 10 h [19]. RSV undergoes considerable phase I and phase II metabolism in the liver, resulting in the formation of glucuronic acid and sulfate conjugates, found in the b Szym loodstream, that preserve biological function [16]. Phase II sulfation and glucuronidation are catalyzed by sulfotransferase and uridine 5′-diphospho-glucuronosyltransferase enzymes, respectively [20]. RSV phase I hydroxylation by CYP1B1 produces piceatannol, characterized by higher antioxidant properties [20]. In human studies using a single oral dose (25 mg), the free RSV blood peak was around 10 ng/mL within 2 h. Metabolite concentrations reached 500 ng/mL, suggesting that also conjugates have biological effects [21]. Free RSV is about 90% bound to plasma proteins, representing a reservoir [18]. RSV administration is generally well tolerated by healthy individuals, and its use in humans is considered safe in vivo [22].

## 2. Platelet Hyperactivation and Aspirin Resistance in T2DM

Platelets are anucleate cell fragments abundant in the bloodstream (150–400 × 10^9^/L), generated by the megakaryocyte in the bone marrow [23]. Platelets express diverse receptors and ligands and contain several organelles (mitochondria, lysosomes, and alpha and dense granules) and specialized canalicular systems. As key players in hemostasis, upon vascular injury, platelets transition from a quiescent to an activated state and adhere to the exposed subendothelial matrix through a multistep process related to the shear conditions of blood flow [24]. Under high shear conditions, platelets are initially captured by VWF through GPIb binding, which enables subsequent firm adhesion to collagen via GPVI and integrin α_2_β_1_ [24]. RSV has been reported to interfere with both VWF-mediated platelet tethering and collagen-dependent stable adhesion [21]. Following adhesion, inside-out signaling triggered by agonists such as thrombin, ADP, and thromboxane A_2_ (TXA_2_) or adhesive proteins drives platelet aggregation by the conformational activation of GPIIb/IIIa (integrin αIIbβ3) [25]. Upon activation, αIIbβ3 shifts from a low- to high-affinity state that binds fibrinogen, VWF, and other ligands (e.g., vitronectin, fibronectin). Activated platelets become cross-linked via fibrinogen and VWF, linking GPIIb/IIIa receptors, leading to aggregate formation. The activated platelet surface promotes the assembly of coagulation factors, stabilizing the developing thrombus [24]. Alterations in platelet indices such as mean platelet volume (MPV), platelet distribution width (PDW), platelet-large cell ratio (P-LCR), and plateletcrit (PCT) have been reported in type 2 diabetes mellitus (T2DM), where they have been proposed as potential biomarkers of poor glycemic control [26]. In T2DM, platelets are hyperreactive, compared to controls [27,28]. Basal platelet activation is similar; however, the stimulated activation is significantly enhanced, which limits the effectiveness of aspirin [29,30]. Aspirin irreversibly blocks cyclooxygenase-1 (COX-1) by acetylating its serine-529 [14] preventing the conversion of arachidonic acid (AA) to thromboxane A2 (TXA2), a potent platelet aggregation agonist. Aspirin resistance (AR) in patients with diabetes is a clinical phenomenon empirically defined as a condition where the conventional dose of aspirin does not sufficiently suppress platelet aggregation [29]. Although AR can be due to other factors, such as lack of adherence to therapy, reduced bioavailability, or interactions with medications, a systematic review discussed AR prevalence in diabetic patients, which is higher than in other populations at cardiovascular risk [29]. AR has been studied more in T2DM than in T1DM. A classic ambulatory study characterized platelets in T1DM patients and found a lab-defined AR phenotype in T1DM associated with female sex, corresponding to a maladaptive phenotype with increased basal activity and hyperactivation upon stimulation [31]. Different direct and indirect laboratory assays are utilized to measure platelet functional AR. Serum thromboxane B_2_ (sTXB_2_) is the most specific pharmacodynamic marker of platelet COX-1 activity. High serum sTXB_2_ levels suggest inadequate platelet inhibition [32]. Test of urinary 11-dehydro-TXB_2_ reflects TXA_2_ but is less specific. Point-of-care test VerifyNow^®^ Aspirin measures platelet response to aspirin using an arachidonic acid agonist to measure their ability to aggregate (results are expressed in Aspirin Reaction Units) [33]. The PFA-100 Indirect assay is another point-of-care assay that evaluates platelet reactivity in high-shear flow by measuring the time it takes for a platelet plug to occlude a small aperture in a membrane-coated cartridge (Closure Time CT) [34]. The concordance between assays is low due to a lack of standardization. Therefore, it is recommended to use at least one direct test and one functional test to investigate AR in T2DM [35]. Several features of T2DM can impair aspirin ability to suppress platelet aggregation. A systematic review comparing the characteristics of AR versus non-AR T2DM patients revealed that AR patients tend to be younger, have higher fasting glucose and HbA1c levels, higher rates of dyslipidemia, and a higher body mass index (BMI). However, no significant differences were observed in gender, comorbidities, or concurrent medications between the two groups [29]. Hyperglycemia induces nonenzymatic glycation of surface platelet proteins, decreasing membrane fluidity, and increasing protein kinase C activation [36]. In T2DM, inflammation enhances platelet phosphatidylserine (PS) exposure, thereby promoting increase expression of the surface glycoproteins Ib and IIb/IIIa [28], of Fcγ receptor type IIa (FcγRIIa) and factor V^a^ binding [37]. Diabetes is associated with systemic inflammation and oxidative stress that may contribute to increased platelet reactivity [38]. Patients with T2DM exhibit constitutively activated P2Y_12_ receptor expression, causing ADP-induced platelet hyperreactivity [39]. Hypertriglyceridemia due to elevated VLDL (common in T2DM) correlates with AR, partly linked to apolipoprotein E. Guidelines still recommend low-dose aspirin (75–100 mg daily) for secondary prevention in diabetes, but individualized use for primary prevention. Routine twice-daily (BID) dosing for T2DM patients is not recommended, due to a lack of evidence (Diabetes Care 2024 guidelines) [40]. A small pharmacodynamic (PD) study trial on T2DM patients without CVD randomized in a three-way crossover design to a two-week treatment showed that 100 mg BID low-dose aspirin reduced platelet reactivity better than 100 mg once a day (QD) and numerically more than 200 mg QD. Clinical outcome trials evaluating primary CVD prevention with aspirin in Type 2 diabetes may need to consider using a more frequent dosing schedule [41]. No outcomes from large, randomized trials yet demonstrate that BID (or higher dose) improves CVD in T2DM compared with standard QD dosing. The Ongoing ANDAMAN trial on adult (type 1 or type 2) patients with DM or AR admitted to the intensive cardiac care unit plans to evaluate the superiority of twice-daily compared to once-daily aspirin in patients with DM or AR during a follow-up of 18 months after acute coronary syndrome [42]. The ADAPTABLE study, a large open label, multicentric trial, enrolled patients with DM and concomitant CVD and randomized them to 81 mg or 325 mg of daily aspirin. No difference was found for the daily aspirin dosing strategies for patients with DM in the primary outcomes (death, myocardial infarction, or hospitalization for stroke) or safety outcomes (major bleeding) [43].

### Endothelial Dysfunction in Type 2 Diabetes

In T2DM, AR results from a combination of internal platelet changes and external factors that increase the risk of thrombosis [12,23]. In the T2DM dysmetabolic and pro-oxidant milieu, hyperreactive platelets establish a crosstalk with endothelial cells [30], contributing to endothelial dysfunction, a hallmark of T2DM. The intact endothelium, a monolayer lining the inner surface of the vascular lumen, maintains an antithrombotic condition by producing nitric oxide (NO) and prostacyclin (PGI_2_), which retard platelet activation by increasing intraplatelet concentrations of cyclic guanosine- and adenosine-monophosphate [44]. Vascular oxidative stress reduces NO and PGI_2_ availability [45]. Contributing to platelet hyperreactivity and endothelial activation [46], increasing the release of VWF, which promotes platelet adhesion and enhanced platelet consumption and turnover. The latter in turn causes newly formed immature platelets with uninhibited COX-1 to enter the circulation [29]. The American Diabetes Association (ADA), which provides the current clinical practice recommendations for DM care [40], recommends standard low-dose aspirin for secondary prevention, but individualized use for primary prevention, as the bleeding risk may outweigh the cardiovascular benefit of aspirin, and because T2DM patient platelets may not respond adequately to aspirin therapy [47]. Antiplatelet bioactive compounds in food may represent an early intervention to prevent AR and thus may prevent T2DM, significantly impacting T2DM complications [48].

## 3. Clinical Use of Resveratrol in Diabetes

RSV displays antibacterial effects against various pathogens (*Campylobacter*, *Staphylococcus aureus*, and others), related to its ability to inhibit the ATP synthase, decreasing the bacterial cellular energy [49]. In humans RSV has numerous promising therapeutic properties, such as antioxidant, anti-inflammatory, endothelial protective, antitumor, anti-adipogenic, and antidiabetic, and has been suggested to be able to modulate AR states [16,50,51,52]. RSV inhibits platelet aggregation by suppressing thromboxane A_2_ (TXA_2_) synthesis through COX-1 inhibition, improves glucose homeostasis, decreases insulin resistance, diminishes AR, protects pancreatic β-cells, and increases GLUT4 and GLUT2 levels [51]. RSV improves glycemic control and insulin resistance in DM by enhancing glucose uptake, promoting GLUT4 expression and translocation, and activating the NAD+-dependent histone deacetylase Silent Information Regulator 1 (Sirtuin1), which inhibits Forkhead transcription factor O1 (FOXO1) expression, exerting protective effects on mitochondrial dysfunction, one of the main drivers of T2DM [50,51]. By attenuating oxidative stress, RSV exerts protective effects in diabetic retinopathy and DM macrovascular complications [53]. RSV modulates several dysregulated metabolic and signaling pathways, such as 5′ AMP-dependent protein kinase (AMPK) and Sirtuin1, protecting pancreatic β-cells and lowering the levels of circulating free fatty acids (FFAs), reducing FFA-induced lipotoxicity. At the platelet level, RSV preserved their ability to aggregate, reducing post-storage prothrombotic action [54]. Ex vivo studies on platelets demonstrated that RSV reduces platelet oxygen consumption, aggregation, and TXA_2_ release, reflecting inhibition of platelet metabolic hyperactivity [55]. RSV acts on several intracellular signaling cascades implicated in platelet activation, including PI3K/Akt, PKC, and MAPK pathways, and promotes NO and cyclic GMP (cGMP) signaling, modulating calcium mobilization and granule secretion [56,57]. Studies on the beneficial effects of RSV on diabetes in DM have been mostly conducted on animal models or in vitro (reviewed in [50]). Some clinical studies confirm the notion that RSV supplementation reduces systemic inflammation and oxidative stress, improving lipid and endothelial profiles in DM, while others do not find significant results (see Table 1) [58,59,60,61,62,63,64,65,66,67].

**Figure 1:**
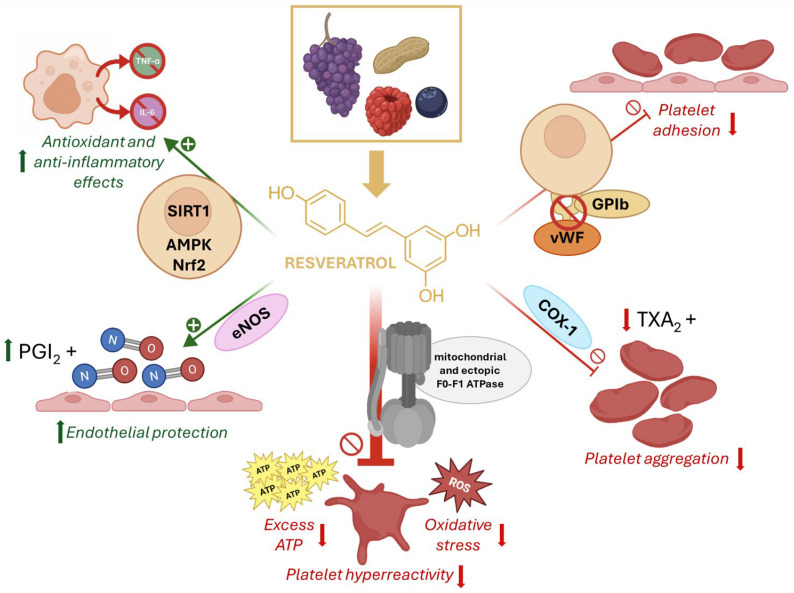
Chemical structure and mechanisms of resveratrol in platelet hyperactivation. Resveratrol (RSV), a natural polyphenol found in grapes, peanuts, and berries, exerts antioxidant and anti-inflammatory effects through activation of SIRT1, AMPK, and Nrf2 pathways. It enhances endothelial protection by stimulating nitric oxide synthase (eNOS) and prostacyclin (PGI_2_) release. RSV inhibits platelet adhesion by interfering with von Willebrand factor (vWF) binding to GPIb, and reduces platelet aggregation by suppressing cyclooxygenase-1 (COX-1)–mediated thromboxane A_2_ (TXA_2_) formation. Importantly, RSV directly modulates mitochondrial and ectopic F_0_F_1_-ATP synthase, reducing excess ATP production and oxidative stress, thereby attenuating platelet hyperreactivity.

Nonetheless, a randomized meta-analysis on patients with T2DM concluded that RSV supplementation led to reductions in C-reactive protein levels, lipid peroxidation markers, and oxidative stress [68]. Additionally, RSV improved resistance to oxidative stress by promoting the expression of antioxidant enzymes such as glutathione peroxidase and catalase, thereby exerting beneficial effects on inflammation and oxidative stress [68]. A single-blind, randomized controlled clinical trial on elderly T2DM patients, assessing a 6-month treatment period with RSV, reported improved blood glucose control, inflammation, insulin resistance, and renal function [69]. The modulation of the same molecular targets, including also endothelial NO synthase (eNOS) exerts protective effects of RSV on the endothelium [57]. Specifically, RSV may modulate VWF binding to platelet glycoprotein Ib (GPIb), a critical interaction under high shear stress that initiates platelet adhesion to the endothelium, and thrombus formation [55]. As antiplatelet drugs like aspirin are not recommended for primary prevention, RSV may be a viable alternative to prevent AR, particularly in patients with T2DM. RSV pleiotropic actions can favorably affect AR (Figure 1) [50].

## 4. The F_1_F_o_-ATP Synthase

The F_1_F_o_-ATP synthase (ATP synthase), or Complex V, is a protein complex that couples the proton gradient generated by the ETC Complexes I–IV to ATP production [70]. The ATP synthase is a key enzyme of the oxidative phosphorylation (OxPhos) pathway [71]; it employs a transmembrane protonmotive force as a source of energy to drive a mechanical rotary catalytic mechanism that synthesizes ATP from ADP and phosphate. The ATP synthase structure comprises a F_o_ moiety, constituted by a membrane-embedded rotor ring (8–14 c-subunits) and the a-subunit that allows protons to flow down their electrochemical gradient, and a catalytic F_1_ moiety protruding into the matrix (α_3_β_3_ hexamer with central γ, δ, ε subunits). The F_1_ can hydrolyze ATP. The inhibitory factor 1 (IF1) binds the F_1_ domain to prevent ATP waste when the mitochondrial membrane potential collapses [72]. Genetic defects in the ATP synthase are associated with mitochondrial disorders, a group of genetic conditions that impair the OxPhos [73]. The ATP synthase is found in the membranes of mitochondria, bacteria, and chloroplasts; however, biochemical, proteomic, and imaging studies have shown that it is also present in ectopic locations [74]. An ectopic ATP synthase is expressed on the plasma membrane (in this case, defined ecto-ATP synthase) of many cancer cells [75,76,77,78]. The ectopic ATP synthases not only carry out the synthesis of extracellular ATP, such as on the neuronal surface [79], human umbilical vein endothelial (HUVEC) cells [79,80] and hepatocytes [81], but also participate in numerous cellular functions [82]. Citreoviridin, an ATP synthase inhibitor, showed cytotoxic effects on NSCLC cells expressing an ecto-ATP synthase [76]. The ectopic ETC complexes coupled to the synthase carry out an OxPhos in rod outer segment (OS) disks and myelin sheath [83], exosomes and microvesicles [84,85], and platelets [86,87]. Our previous data showed that human platelets exhibit an extra-mitochondrial OxPhos, representing an additional source of the chemical energy needed to support activation [86,87]. Immunofluorescence analysis showed the co-localization with calnexin (a marker of endoplasmic reticulum, ER) of subunit II of Cytochrome *c* Oxidase (COXII) encoded by mitochondrial DNA and ATP synthase, but not TIM, suggesting that in platelets, the extra-mitochondrial OxPhos could occur in the inner membranes, such as the ER [86]. Western Blot analysis of platelets revealed that the ratio of ATP synthase to TIM (Translocase of the Inner Mitochondrial membrane) signal was approximately two-fold higher in platelets compared with mitochondria [86]. The communication system between organelles, known as mitochondria-associated ER membranes (MAMs), may be involved in the putative transfer of the OxPhos machinery to ectopic locations, primarily the ER [88]. Several proteins tether the mitochondria to the ER, creating dynamic regions that regulate various biological processes, including mitochondrial dynamics. Interestingly, dysregulation of MAMs is associated with the progression of several disorders, particularly diabetes mellitus [88]. A hallmark of platelets is their remarkable metabolic flexibility [87,89,90,91], enabling them to adapt to the continually varying environmental and functional conditions, from a resting state mostly relying on glycolysis to an activated state characterized by a shift to oxidative metabolism [90,91]. Platelet activation triggers an increase in OxPhos sustained enhanced glucose uptake (mediated primarily by glucose transporter 3, GLUT3), which glycolysis alone cannot support [90]. The remarkable ability of platelets to shift their use of substrates, specifically glucose and fatty acids, upon activation could depend on the subcellular compartmentalization of metabolic pathways and the timely redirection of resources [87,90]. The extra-mitochondrial OxPhos capacity of platelets would represent a compartment able to utilize glucose to fuel an oxidative metabolism outside the mitochondria that would readily supply ATP to the cell [87]. However, the extra-mitochondrial OxPhos can have both beneficial and detrimental effects, being the primary source of reactive oxygen species (ROS) production [92]. Hyperglycemia can enhance OxPhos and contribute to platelet hyperactivation in T2DM [93]. When platelets become hyperactivated in T2DM, increased glucose uptake can overstimulate the extra-mitochondrial OxPhos, leading to a dangerous increase in ROS in the cytosol. In this context, in addition to the numerous beneficial effects of RSV [55], the action of RSV binding to the ectopic ATP synthase F_o_ moiety in the platelets is noteworthy, justifying the use of RSV as an effective antiplatelet therapy. In a streptozotocin-induced T2DM rat model, platelet hyperactivation was associated with increased OxPhos, not observed in the hepatocyte mitochondria [94]. RSV specific antioxidant modulatory action on the ectopic ATP synthase could limit the detrimental oxidative stress production in the platelet cytosol. In fact, the modulation of the ATP synthase by polyphenols has been shown to reduce ROS production by the ectopic ETC [95].

## 5. Resveratrol Inhibition of ATP Synthase and Its Relevance in AR

**Figure 2:**
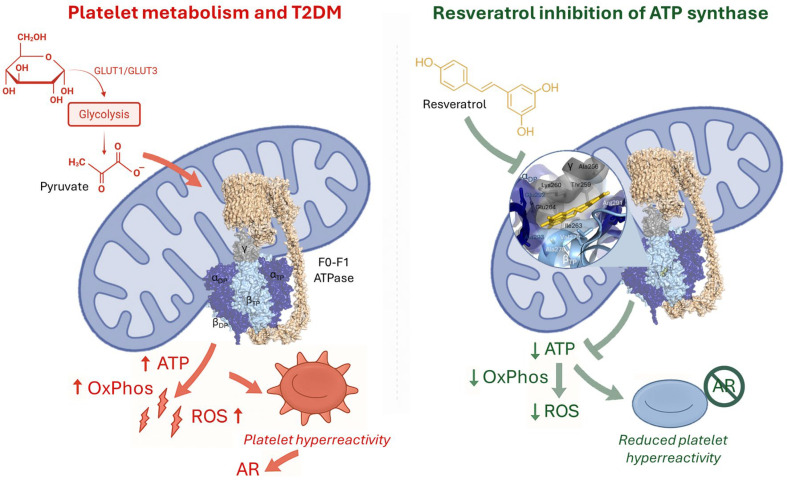
Platelet metabolism in type 2 diabetes mellitus (T2DM) and resveratrol inhibition of ATP synthase. (**Left panel**) In T2DM, increased glucose uptake and glycolysis lead to excessive pyruvate availability, fueling mitochondrial oxidative phosphorylation (OxPhos) via the F_0_F_1_-ATPase complex. This results in excessive ATP and reactive oxygen species (ROS) production, contributing to platelet hyperreactivity and aspirin resistance (AR). (**Right panel**) Resveratrol directly interacts with the ATP synthase complex, reducing OxPhos activity, ATP output, and ROS generation. This inhibition attenuates platelet hyperreactivity and counteracts AR, highlighting its potential as an adjunct antiplatelet therapy in diabetes. The structural model of ATP synthase (PDB: 2JIZ) is shown, with a magnified view illustrating key amino acid residues predicted to interact with resveratrol.

RSV binds and inhibits the mitochondrial ATP synthase, as shown by structural studies [80] that showed a direct interaction of the polyphenol with its F_1_ moiety, which may underlie some of its bioenergetic and signaling effects. The modulation of the ectopic OxPhos by RSV which can bind ATP synthase in the platelets may play a pivotal role in alleviating the ROS production, ultimately counteracting AR. The mitochondrial dysfunction in AR may include the extra-mitochondrial OxPhos. The platelet metabolic microenvironment influences multiple physiological and pathological conditions [86]. Platelets exhibit a distinct metabolic flexibility that allows them to adapt to varying conditions regarding substrate availability and metabolic capacity [87]. While resting platelets mainly utilize anaerobic glycolysis, activated platelets would rely on an extra-mitochondrial OxPhos that utilizes glucose and a mitochondrial OxPhos that utilizes fatty acids [87]. OxPhos over-functioning, such as in the case of chronic hyperglycemia, can lead to excess ROS generation. Hyperglycemia-induced oxidative stress plays a pivotal role in the development of diabetes complications [96]. Intraplatelet glucose concentration mirrors the blood concentrations, and hyperglycemia is an AR cause factor. Upon activation, platelets undergo a rapid uptake of glucose through GLUT3 and switch to an oxidative metabolism, which relies on the ETC, the primary source of ROS [97]. Extra-mitochondrial OxPhos overfunctioning, driven by excess glucose availability, can lead to increased ROS production in the cytosol, which could be a primary contributor to the platelet oxidative stress and AR. Excessive OxPhos activity can lead to increased ROS production, both inside the mitochondrial matrix and in the cytosol, due to extra-mitochondrial OxPhos, which contributes to oxidative stress. Elevated ROS and inflammation determine a pro-thrombotic environment. The modulation of platelet ectopic OxPhos by RSV may play a pivotal role in counteracting oxidative stress, ultimately alleviating AR [98]. This hypothesis is consistent with the data showing that the ATP synthase as the molecular target of Chromium (III), a nontoxic form of chromium. In hepatic cells (HepG2), Cr^3+^ binds the ATP synthase β subunit, the catalytic subunit of the ATP synthase, abolishing its catalytic activity in a dose-dependent manner, which ameliorates hyperglycemia [99]. Also, it was proposed that elevated serum IF1 levels, protective against CVD may act by inhibiting the ecto-ATP synthase [100]. These data suggest that the inhibition of ATP synthase by RSV might be important in its action against AR (Figure 2).

## 6. Conclusions

RSV exhibits strong mechanistic rationale and preclinical evidence as a potential adjunctive therapy for T2DM. However, its clinical application as an antiplatelet treatment for patients with AR is still in the early stages. To fully understand the protective effects of RSV, its inhibitory action on the platelet extra-mitochondrial ATP synthase, as well as the ectopic ATP synthase found in the plasma membrane of endothelial cells, should be taken into account. Modulating these components, along with the ETC associated with them, can lead to a reduction in AR, endothelial activation, and ultimately inflammation. Future randomized controlled trials involving T2DM patients with confirmed AR may help clarify optimal dosages and treatment duration. There are some limitations to the clinical implementation of RSV for AR. These include its poor bioavailability due to rapid metabolism, the lack of sufficient data regarding AR in T2DM patients, uncertainties surrounding dose–response relationships, and the potential for interactions with antiplatelet or anticoagulant therapies that could increase the risk of bleeding.

## Figures and Tables

**Table 1:** Clinical Studies of Resveratrol in T2DM (Human Trials) Randomized or controlled clinical trial, including study type, study population, sample size, and potential efficacy.

Citation	Design	Study Population	Dose & Duration	Primary Endpoints	Main Findings
Mahjabeen et al. [59]	Randomized, double-blind, placebocontrolled, parallel-group	n = 110 randomized; 94 completed; adults with T2D on OHDs	Resveratrol 200 mg/day, 24 weeks	Fasting glucose, insulin, HOMA-IR; hs-CRP, TNF-α, IL-6; MDA; circulating microRNAs	Significant reductions in glucose, insulin, HOMA-IR, hs-CRP, TNF-α, IL-6, and MDA vs. placebo; favorable miRNA changes; no major AEs
Bo et al. [58]	Randomized, double-blind, placebo-controlled, 3-arm	n = 192; adults with T2D	Resveratrol 40 mg/day or 500 mg/day vs. placebo, 6 months	CRP; metabolic parameters incl. HbA1c, glucose, insulin, BP, lipids	No significant effects on CRP or metabolic profile vs. placebo (dose-dependent trend only for CRP); well tolerated
Bo et al. [60]	Randomized, double-blind, placebo-controlled, 3-arm (secondary analysis)	n = 192; adults with T2D	Resveratrol 40 or 500 mg/day vs. placebo, 6 months	Pentraxin-3 (PTX3) and Total Antioxidant Status (TAS)	Dose-dependent increase in PTX3 and TAS vs. placebo; clinical significance uncertain
Timmers et al. [61]	Randomized, double-blind, crossover	n = 17; men with well-controlled T2D	Resveratrol 150 mg/day, 30 days per period	Hepatic & peripheral insulin sensitivity (clamp); secondary: muscle mitochondrial function	No improvement in insulin sensitivity; ex vivo muscle mitochondrial function increased; possible interaction with metformin
Thazhath et al. [62]	Randomized, double-blind, placebo-controlled, crossover	n = 14; diet-controlled T2D	Resveratrol 500 mg twice daily, 5 weeks per period	GLP-1 secretion, gastric emptying; secondary: HbA1c, glucose, weight, energy intake	No effect on GLP-1, gastric emptying, or glycemic control vs. placebo
Abdollahi et al. [63]	Randomized, double-blind, placebo-controlled	n = 71; overweight adults with T2D (BMI 25–30)	Resveratrol 1000 mg/day, 8 weeks	Fasting glucose, insulin, HOMA-IR; lipids; body composition	Decreased fasting glucose, insulin, HOMA-IR; increased HDL; no changes in anthropometrics
Seyyedebrahimi et al. [64]	Randomized, double-blind, placebo-controlled	n = 48; adults with T2D	Resveratrol 800 mg/day, 8 weeks	Oxidative stress markers in blood PBMCs (MDA, antioxid. enzymes)	Significant antioxidant effects vs. placebo;
Hoseini et al. [65]	Randomized, double-blind, placebo-controlled trial	n = 56; T2D with coronary heart disease	Resveratrol 500 mg/day, 4 weeks	Metabolic status (glycemic indices, lipids), inflammatory markers	Short-term improvements in metabolic status vs. placebo
García-Martínez et al. [66]	Randomized, three-arm clinical trial	n = 97 (EG1000 n = 37; EG500 n = 32; placebo n = 28)	Resveratrol 500 or 1000 mg/day vs. placebo, 6 months	Oxidative stress panel; SIRT1	Improved antioxidant indices and SIRT1 with 1000 mg/day; no significant changes in glucose/HbA1c
García-Martínez et al. [67]	Randomized, double-blind, placebo-controlled trial	n = 71 adults with T2D	Resveratrol 500 mg/day, 6 months	Oxidative stress and inflammatory biomarkers (MDA, SOD, CAT, GPx, IL-6, TNF-α)	Resveratrol significantly reduced MDA, IL-6, and TNF-α and increased SOD, CAT, and GPx vs. placebo; improved antioxidant and inflammatory profile; well tolerated.

## Data Availability

No new data were created or analyzed in this study. Data sharing is not applicable to this article.

## Abbreviations

The following abbreviations are used in this manuscript:

CVD	Cardiovascular Disease
DM	Diabetes mellitus
ETC	Electron Trasport Chain
RSV	Resveratrol
AR	Aspirin Resistance
T2DM	Type 2 Diabetes mellitus
TXA_2_	Thromboxane A_2_
